# Fingerprinting seizure outcome after temporal lobe surgery using preoperative connectomic mapping

**DOI:** 10.1093/braincomms/fcac158

**Published:** 2022-06-14

**Authors:** Simon S Keller

**Affiliations:** Department of Pharmacology and Therapeutics, Institute of Translational Medicine, University of Liverpool, Liverpool, UK

## Abstract

This scientific commentary refers to ‘Presurgical temporal lobe epilepsy connectome fingerprint for seizure outcome prediction’ by Morgan *et al*. (https://doi.org/10.1093/braincomms/fcac128) in *Brain Communications*

This scientific commentary refers to ‘Presurgical temporal lobe epilepsy connectome fingerprint for seizure outcome prediction’ by Morgan *et al*. (https://doi.org/10.1093/braincomms/fcac128) in *Brain Communications*.

Medically refractory epilepsy continues to be a major health burden. It is associated with uncontrolled recurrent epileptic seizures and a myriad of social, cognitive, affective and psychiatric co-morbidities, which deleteriously impact on a patient’s quality of life. In carefully selected patients, neurosurgical intervention can be highly therapeutic. Temporal lobe surgery continues to be the most frequently administered surgery for refractory focal epilepsy. It is commonly associated with a postoperative improvement, as most patients experience a significant reduction in postoperative seizures and no surgery-related complications. The risks associated with epilepsy surgery are substantially lower than the risks associated with uncontrolled epileptic seizures, particularly tonic-clonic seizures.^1^

Despite that most patients benefit from surgery, approximately half will not attain complete postoperative seizure freedom, and the mechanisms driving postoperative seizures remain unknown in many patients. The complete elimination of seizures (and no surgically induced morbidity) is the perfect outcome and what every patient and treating clinician strives for. Ahead of potential surgery, patients and clinicians would benefit from knowing what the likely seizure outcome will be to manage expectations and potentially re-route patients to alternative treatment pathways if resective surgery is unlikely to be curative. There have been many reports of factors associated with a favourable outcome after temporal lobe surgery. These include clinically related factors such as absence of secondary generalized tonic-clonic seizures, a lesion (in particular, hippocampal sclerosis) on MRI, and pure temporal lobe epilepsy (TLE) as opposed to temporal-plus epilepsy,^2,3^ surgical factors such as absence of hippocampal remnant and an increased extent of surgical resection,^3,4^ and histological factors including absence of atypical forms of hippocampal sclerosis.^5^

There is also an accumulating number of structural and functional MRI studies that have indicated that outcome after temporal lobectomy is superior when imaging abnormalities are constrained to ipsilateral temporal lobe regions and inferior when structural and functional alterations extend across wider areas, particularly throughout the limbic system^6^ and impact on thalamo-cortical networks.^7^ This is an evolving area of research grounded in group-wise statistical comparisons (e.g. retrospective comparison of preoperative MRI data in patients who were rendered seizure free and those who continued to experience postoperative seizures). There have been recent attempts to prognosticate patients using artificial intelligence approaches, typically applied to preoperative MRI connectome data.^8^ Studies utilizing connectomic approaches—that is, approaches that model whole-brain structural and/or functional connections—is intuitive given that epilepsy is the archetypal network disorder. Seizures start within and propagate throughout neural networks, and modelling brain structural and functional networks using connnectomic approaches have the potential to unlock imaging biomarkers of treatment outcome. The trick now is to bring connectomic analysis to the clinic for individual patients by developing personalized prognostic markers—or connectomic fingerprints.

Morgan *et al*.^9^ characterized the preoperative whole-brain connectome fingerprint—derived from both structural and functional MRI data—of a subgroup of patients with unilateral TLE who were surgically rendered seizure free. The authors hypothesized that these patients—the ‘model group’—have a unique connectome fingerprint that makes them amenable to successful temporal lobe surgery (i.e. surgery leading to complete seizure freedom). The crux of the approach then assumes that patients with connectome fingerprints that are dissimilar—or have increased structural and functional distance—from the model group are less likely to achieve postoperative seizure freedom. This is exactly what the authors report; patients with Engel outcome classification of III–IV had significantly greater connectivity distances than patients with Engel I–II outcomes, but only when both structural and functional connectomes were incorporated. A receiver operating characteristic (ROC) curve analysis of total distance had 100% sensitivity and 90% specificity for the classification of outcome. There was also no relationship between total distance to the TLE connectome fingerprint and patient clinical parameters.

There are several advantages of the method described by Morgan *et al*.^9^ One is the potential uptake in clinical environments. All too frequently do advanced imaging methods distance themselves from clinical implementation given the specialized and technical nature of data pre-processing, analysis and interpretation. Provided that the appropriate MRI data are acquired, and a standardized and user-independent pathway for data processing incorporated, the interpretation of findings for individual patients is theoretically straightforward using the Morgan *et al*. approach and not open to convoluted and difficult to interpret findings as seen using other approaches (e.g. graph theory). Using connectomic fingerprinting, an individual patient’s whole-brain connectivity profile can be automatically computed and compared with the TLE fingerprint, and the degree of similarity and distance can be visualized and quantified. As the authors state, ‘If distances are higher than the other patients with Engel I–II outcome, more localization testing or other treatments may be considered’. An additional advantage is that the approach may account for inter-individual differences driving seizure recurrence after surgery, whereas many studies using conventional group-based comparisons assume a common reason for recurrence. It is likely that the reasons for continued seizures after surgery are multifactorial and differ between patients.

As with all approaches, there remain some methodological issues to consider, which the authors commendably discuss. The results are limited to prediction of a small group of patients with the worst outcomes (Engel III–IV), who do represent a relative minority of patients after temporal lobe surgery. Moreover, outcome was assessed at 1 year after surgery and as time goes on, more patients generally relapse, and outcome is dynamic as some patients’ lapse-remit.^10^ Therefore, and as the authors recommend, multicentre trials on larger cohorts of patients with longer term outcomes are encouraged. Also, given that different fingerprints were found for structural and functional connectivity, the proposed method is predictive of outcome only when both measures are combined, which necessitates the acquisition of both diffusion and resting-state functional MRI data ahead of surgery. Diffusion MRI may frequently be acquired at this time (e.g. tractography for visualization of eloquent tracts ahead of surgery) but resting-state functional MRI is still a research MRI sequence not routinely incorporated into preoperative MRI protocols. Finally, it will be important to determine whether similar approaches are effective in predicting outcome in patients with other kinds of refractory focal epilepsy, particularly given the sharp decrease in the number of temporal lobectomies for TLE with hippocampal sclerosis and rise in extratemporal surgeries.^3^

For those interested in the connectomic fingerprint approach, the authors freely provide the algorithms used to develop patient fingerprints via GitHub (see paper for link).

The technological advancements that allow us to eloquently visualize and measure brain structure, connectivity and function *in vivo* have not yet led to a noticeable increased number of patients that experience seizure freedom after epilepsy surgery. Seizure outcome rates after temporal lobe surgery have remained relatively stable for decades. For the increasingly sophisticated neuroimaging methods to impact personalized medicine and surgery innovative ways of individualizing imaging phenotypes need to be developed and trialled that can seamlessly be incorporated into routine clinical evaluation and administered by treating clinicians. Those that are easy to compute, interrogate and interpret stand the greatest chance of being embedded into clinical pathways.

## Data Availability

Data sharing is not applicable to this article as no new data were created or analysed.
